# Engagement With an Internet-Administered, Guided, Low-Intensity Cognitive Behavioral Therapy Intervention for Parents of Children Treated for Cancer: Analysis of Log-Data From the ENGAGE Feasibility Trial

**DOI:** 10.2196/67171

**Published:** 2025-01-28

**Authors:** Christina Reuther, Louise von Essen, Mudassir Imran Mustafa, Markus Saarijärvi, Joanne Woodford

**Affiliations:** 1 CIRCLE - Complex Intervention Research in Health and Care Department of Women's and Children's Health Uppsala University Uppsala Sweden; 2 Department of Neurobiology, Care Sciences and Society Karolinska Institute Stockholm Sweden

**Keywords:** childhood cancer survivor, cognitive behavioral therapy, engagement, internet-administered intervention, log-data, parents

## Abstract

**Background:**

Parents of children treated for cancer may experience psychological difficulties including depression, anxiety, and posttraumatic stress. Digital interventions, such as internet-administered cognitive behavioral therapy, offer an accessible and flexible means to support parents. However, engagement with and adherence to digital interventions remain a significant challenge, potentially limiting efficacy. Understanding factors influencing user engagement and adherence is crucial for enhancing the acceptability, feasibility, and efficacy of these interventions. We developed an internet-administered, guided, low-intensity cognitive behavioral therapy (LICBT)–based self-help intervention for parents of children treated for cancer, (EJDeR [internetbaserad självhjälp för föräldrar till barn som avslutat en behandling mot cancer or internet-based self-help for parents of children who have completed cancer treatment]). EJDeR included 2 LICBT techniques—behavioral activation and worry management. Subsequently, we conducted the ENGAGE feasibility trial and EJDeR was found to be acceptable and feasible. However, intervention adherence rates were marginally under progression criteria.

**Objective:**

This study aimed to (1) describe user engagement with the EJDeR intervention and examine whether (2) sociodemographic characteristics differed between adherers and nonadherers, (3) depression and anxiety scores differed between adherers and nonadherers at baseline, (4) user engagement differed between adherers and nonadherers, and (5) user engagement differed between fathers and mothers.

**Methods:**

We performed a secondary analysis of ENGAGE data, including 71 participants. User engagement data were collected through log-data tracking, for example, communication with e-therapists, homework submissions, log-ins, minutes working with EJDeR, and modules completed. Chi-square tests examined differences between adherers and nonadherers and fathers and mothers concerning categorical data. Independent-samples *t* tests examined differences regarding continuous variables.

**Results:**

Module completion rates were higher among those who worked with behavioral activation as their first LICBT module versus worry management. Of the 20 nonadherers who opened the first LICBT module allocated, 30% (n=6) opened behavioral activation and 70% (n=14) opened worry management. No significant differences in sociodemographic characteristics were found. Nonadherers who opened behavioral activation as the first LICBT module allocated had a significantly higher level of depression symptoms at baseline than adherers. No other differences in depression and anxiety scores between adherers and nonadherers were found. Minutes working with EJDeR, number of log-ins, days using EJDeR, number of written messages sent to e-therapists, number of written messages sent to participants, and total number of homework exercises submitted were significantly higher among adherers than among nonadherers. There were no significant differences between fathers and mothers regarding user engagement variables.

**Conclusions:**

Straightforward techniques, such as behavioral activation, may be well-suited for digital delivery, and more complex techniques, such as worry management, may require modifications to improve user engagement. User engagement was measured behaviorally, for example, through log-data tracking, and future research should measure emotional and cognitive components of engagement.

**Trial Registration:**

ISRCTN Registry 57233429; https://doi.org/10.1186/ISRCTN57233429

## Introduction

### Background

The digital delivery of psychological interventions, such as cognitive behavioral therapy (CBT), particularly internet-administered cognitive behavioral therapy (iCBT), has rapidly grown over the past 2 decades [1]. iCBT interventions have shown to be effective in reducing symptoms of depression [2] and anxiety [3,4], with outcomes comparable with traditional face-to-face CBT when professional guidance is included as a component [5,6]. Flexibility, convenience [7], and the ability to overcome logistical and financial barriers [8], alongside the potential to reduce the stigma associated with seeking mental health treatment [8,9], are significant advantages of iCBT. In addition, given the lack of available therapists, iCBT interventions represent a potentially low-cost alternative to disseminating psychological interventions to a wider audience [10].

Despite these advantages, how users engage with digital interventions, including iCBT, is uncertain [11,12]. User engagement, which can be defined as how and to what extent users actively use digital interventions, is associated with the efficacy of iCBT interventions [13,14]. Low or inconsistent user engagement with digital interventions is closely associated with common issues such as low uptake (ie, a smaller-than-expected number of users participating in and benefiting from the intervention), variable usage (ie, users engaging with the intervention inconsistently or unevenly), and high attrition rates (ie, users drop out of the intervention) [15,16].

A related concept to user engagement is “adherence,” referring to whether an intervention is used in the way it was designed to maximize efficacy [17]. Adherence may be based on a minimal level of user engagement considered necessary to result in changes to intended outcomes, that is, a minimum treatment dose [14]. Although adherence to digital interventions is an evolving concept [18], the total number of completed sessions has been found to predict treatment response in iCBT interventions [19]. However, knowledge concerning the causal pathways between user engagement, adherence, and efficacy remains limited [20]. Furthermore, there is insufficient understanding of the potential association between sociodemographic factors, for example, age, gender, sex, and clinical characteristics (ie, depression or anxiety symptoms), and user engagement and adherence [19]. Adherence rates also vary between studies, with a review of adherence to iCBT interventions reporting adherence rates ranging from 6% to 100% [16]. Furthermore, high attrition rates, particularly in unguided iCBT interventions, present additional challenges [21], potentially resulting in biased treatment effects [22] and limiting generalizability [23].

Although user engagement is associated with treatment response [18], there remains a lack of clarity on how to conceptualize, define, and measure user engagement within the context of digital interventions [11,12,20]. Engagement is considered multidimensional [24,25] and can include to what extent an intervention is used, for example, by log-data tracking to quantitatively capture digital intervention usage [20], as well as the user’s subjective experience, for example, affect, attention, and interest [25]. Therefore, one way to explore user engagement is by analyzing log-data (ie, automatically collected digital records of a participant’s use of an intervention such as session duration, completion rates, and interaction patterns) [26,27] and examining potential relationships between user engagement and participant’s sociodemographic and clinical characteristics [28]. There is also a need to investigate the use of digital interventions to explore how certain aspects of user engagement may relate to adherence [20] and inform the minimal usage required to establish adherence [29].

### This Study

We conducted a single-arm feasibility trial (ENGAGE) [30-32] to examine the acceptability and feasibility of an internet-administered, guided, low-intensity cognitive behavioral therapy (LICBT) intervention for parents of children treated for cancer: EJDeR (Swedish acronym: intErnetbaserad sJälvhjälp för förälDrar till barn som avslutat en behandling mot canceR [Internet-based self-help for parents of children who have completed cancer treatment]) [33-38]. EJDeR was developed alongside parent research partners [38] and is designed for parents of children treated for cancer experiencing symptoms of depression and/or generalized anxiety disorder (GAD) related to their child’s cancer treatment [33-38]. EJDeR is delivered through the Uppsala University Psychosocial Care Programme (U-CARE) portal (Portal), an in-house web-based platform, designed to deliver digital interventions and support the execution of study procedures (eg, online consent and data collection) [38]. EJDeR includes four modules: (1) introduction and psychoeducation (IPE), (2) behavioral activation (BA), (3) worry management (WM), and (4) relapse prevention (RP), and is guided by an e-therapist. Findings from ENGAGE show EJDeR is an acceptable and feasible intervention; therefore, progression to an external pilot randomized controlled trial (RCT) to prepare for the design and conduct of a future superiority RCT is warranted. However, adherence to the minimal treatment dose was 47.9%, marginally under the progression criteria of 50% [31]. In addition, visual inspection of adherence data indicated possible differences in adherence rates by module worked with and between fathers and mothers [31]. Previous research indicates fathers and mothers may engage differently with psychological interventions [39], emphasizing the need to consider gender-specific engagement patterns in digital intervention design. For example, fathers have been found to prefer solution-focused, practical, and time-efficient formats that offer structured guidance and actionable strategies [40]. In contrast, mothers have been found to prefer more emotional and process-oriented intervention content, valuing opportunities for self-reflection and emotional processing [41].

Given these findings, there is a need to conduct a secondary analysis of data from ENGAGE to better understand how participants engaged with the intervention. Results may help inform ways to adapt and modify the intervention to improve adherence rates before proceeding to an external pilot RCT. Further modification of the intervention to improve user engagement and thus adherence may also improve treatment outcomes in a future superiority RCT [31]. Findings may also have wider applicability to other iCBT interventions.

### Study Aims

This study aimed to (1) describe user engagement with the EJDeR intervention, (2) examine whether sociodemographic and baseline clinical characteristics differed between adherers and nonadherers, (3) examine whether depression and anxiety scores differed between adherers and nonadherers at baseline, (4) examine whether user engagement differed between adherers and nonadherers, and (5) examine whether user engagement differed between fathers and mothers.

## Methods

### Study Design

This study is a secondary analysis of data from ENGAGE. The trial protocol [32] and main findings from ENGAGE are reported elsewhere [31].

### Participant Inclusion Criteria

Eligibility criteria included being (1) a parent of a child diagnosed with cancer during childhood (0-18 years) who completed cancer treatment 3 months to 5 years previously (timespan informed by our previous longitudinal research that has identified this as a period of vulnerability for parents [42,43], (2) resident in Sweden, (3) able to read and understand Swedish, (4) able to access email, internet, and BankID (a citizen authentication system used in Sweden), and (5) seeking psychological support related to the child’s cancer. Exclusion criteria included (1) severe and enduring mental health problems, for example, bipolar disorder and psychosis; (2) acute suicidality; (3) misuse of alcohol; street drugs or prescription medication; and (4) currently attending psychological treatment. Participants excluded due to criteria 1-3 were directed to appropriate health care services.

### Participant Recruitment

Parents were recruited into ENGAGE using 2 main approaches. First, personal identification numbers of children were obtained from the Swedish Childhood Cancer Registry (National Quality Registry) and linked to parents’ names and addresses through NAVET, a registry held by the Swedish Tax Agency. Parents were invited to participate by postal invitations using random blocks of 100 until the target number of 50 was reached. Second, advertisements were placed on relevant social media sites and websites of patient organizations and interest groups [31].

An adapted CONSORT (Consolidated Standards of Reporting Trials) diagram for ENGAGE has been published [31]. Recruitment took place over 5 months (July 3, 2020, to November 30, 2020). A total of 72 participants gained access to EJDeR, with 1 excluded shortly after access (severe and enduring mental health difficulty), resulting in a sample of 71.

### Intervention

**Figure 1:**
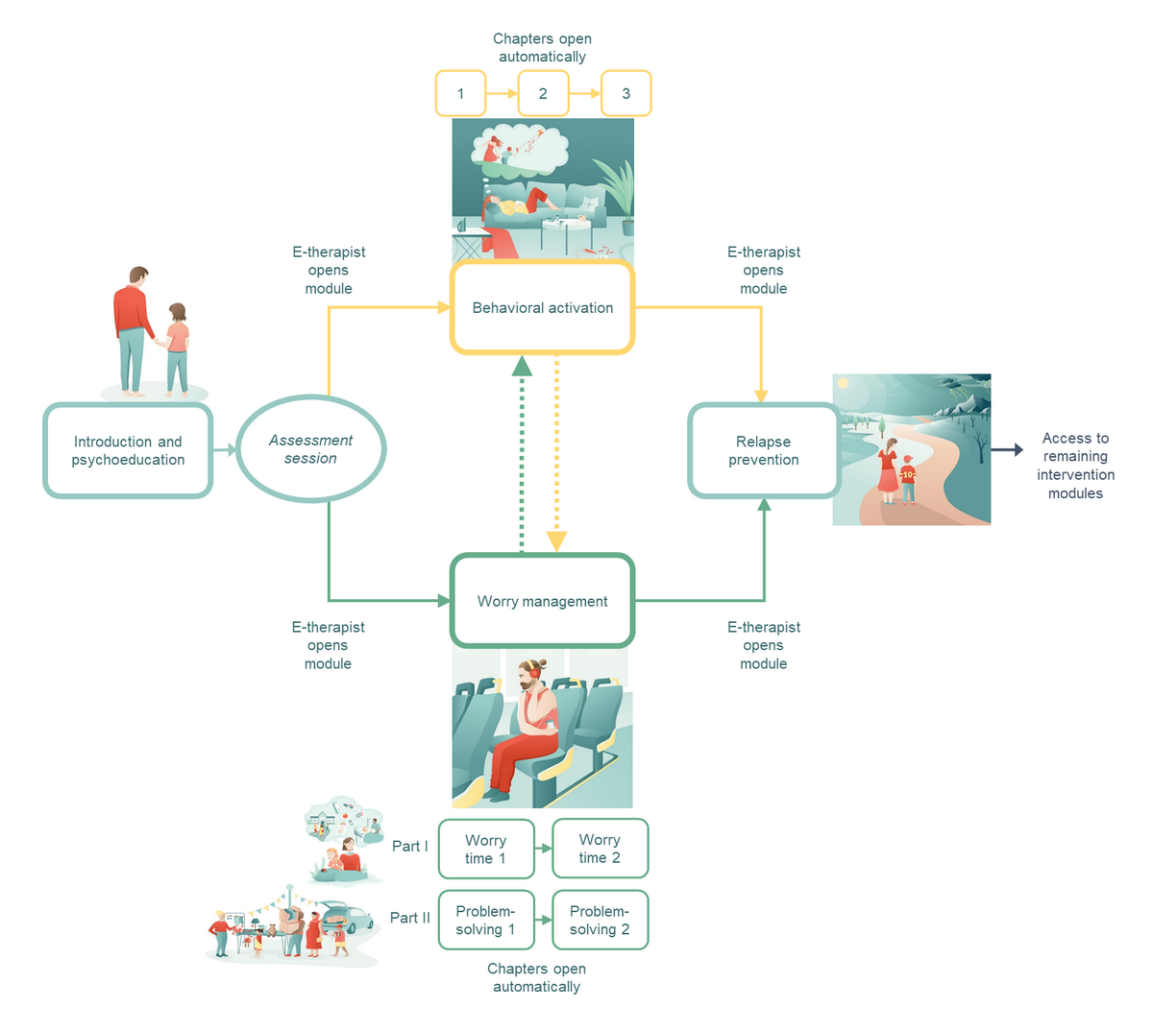
An overview of the structure of EJDeR. Figure from Woodford et al [38] which is published under Creative Commons Attribution 4.0 International License [50].

EJDeR was delivered via the Portal, incorporating text, homework exercises, films, illustrations, and audio content. A detailed description of EJDeR has been published [38], and an overview of the intervention is presented in Figure 1. EJDeR was delivered over 12 weeks and consisted of 4 modules: (1) IPE, (2) BA, (3) WM, and (4) RP. It includes 2 LICBT techniques—BA for depression [44] and WM for GAD [45]. The LICBT clinical protocol for BA has been published elsewhere [44]. Participants are supported to re-engage with pleasurable, necessary, and routine activities they have stopped doing in a gradual and structured way. The full LICBT clinical protocol for WM has also been published elsewhere [45,46]. Participants record worries and categorize worries into 2 types—practical (eg, important and can be solved) and hypothetical (eg, important but have no way of being solved). Participants work with problem-solving for practical worries and worry time for hypothetical worries.

Guidance was provided by e-therapists, who were trained in the competencies required to support LICBT. After being provided with access to EJDeR, participants were invited to attend an initial assessment session (telephone or videoconferencing) with their e-therapist. During the initial assessment, e-therapists used patient-centered interviewing techniques to understand the participant’s main presenting difficulties, provide psychoeducation, introduce the LICBT techniques used in EJDeR, and come to a collaborative decision to allocate 1 of the 2 LICBT modules to first work with, based on the participant’s main difficulty, for example, BA for depression or WM for GAD. E-therapists did not have access to participants’ baseline assessment scores (ie, depression and anxiety symptoms) and therefore allocation to the first LICBT module to work with was not informed by symptom severity at baseline. Subsequently, the e-therapist provided participants with access to the LICBT module. E-therapists provided weekly written messages via the Portal to guide participants in using the intervention [47]. To facilitate engagement with the LICBT modules, weekly homework exercises were either completed on the Portal or printed as a PDF and completed offline. E-therapists also invited participants to attend a midintervention booster session (telephone or videoconferencing). After completing the first LICBT module allocated (BA or WM), participants could choose to work with the remaining LICBT module. After completing either BA or WM, participants were provided with access to RP by their e-therapist.

Persuasive system design elements [48,49] are built into EJDeR to improve user engagement, including tunneling, for example, content is delivered in a predefined step-wise order to guide participants through using EJDeR, tailoring (eg, EJDeR content is personalized to user needs [ie, depression or GAD]), rehearsal (eg, homework exercises are repeated), and liking (eg, use of professional illustrations).

### Intervention Adherence

Adherence to EJDeR was conceptualized as participants engaging with the intervention following a priori–defined minimum treatment dose. Intervention adherers were defined as those (1) attending the initial assessment session with an e-therapist (telephone or videoconferencing), (2) completing the IPE module (2 chapters) and 1 LICBT treatment module (ie, BA [3 chapters] or WM defined as completing an introduction [2 chapters] and completing either problem solving [2 chapters] or worry time [2 chapters]), and (3) attending the midintervention booster session with an e-therapist (telephone or videoconferencing). For a module to be defined as being completed, participants were required to submit all chapters included within each module to their e-therapist on the Portal. Our minimum treatment dose was informed by research suggesting that guidance from a trained professional is associated with larger treatment effects than self-administered LICBT [51,52]. In addition, we considered it important for participants to complete the IPE module to gain an understanding of the CBT rationale and complete all chapters for 1 LICBT technique to enable them to understand and apply the technique to manage their symptoms of depression and/or anxiety. We did not include the submission of weekly homework exercises in our definition of adherence given participants were able to complete on the Portal or print as a PDF and complete offline.

### Measures

#### Sociodemographic Characteristics

Sociodemographic characteristics were collected during a telephone eligibility interview with a licensed psychologist and included age, sex (male or female), relationship status (partner or single), highest level of education (lower secondary, upper secondary, postsecondary nontertiary, tertiary, or PhD), employment status (employed or unemployed), number of children, housing situation (rental, apartment ownership, house ownership, or other), region of birth (Nordic countries, Asia, Europe [excluding Nordic countries], or Africa), previous psychological treatment (yes or no), physical health problem (yes or no), and previous traumatic or difficult life events (yes or no).

#### Baseline Clinical Characteristics

Clinical characteristics were collected via a Portal assessment at baseline. Symptoms of depression were assessed using the 9-item Patient Health Questionnaire (PHQ-9) [53]. Symptoms of anxiety were assessed using the 7-item Generalized Anxiety Disorder scale (GAD-7) [54].

#### User Engagement

##### Log-Data Tracking on the Portal

User action logging was enabled through action metadata management to allow user behavior analysis, including (1) minutes working with EJDeR, (2) number of log-ins, (3) number of days using EJDeR, (4) number of written messages sent to e-therapists, (5) number of written messages sent to participants, (6) total number of modules opened, (7) total number of modules completed, (8) opened modules (IPE and RP), (9) completed modules (IPE and RP), (10) opened first LICBT module allocated (BA and WM), (11) completed first LICBT module allocated (BA and WM), and (12) submitted homework exercises.

##### Data Collected Outside the Portal

Intervention activities outside of the Portal were collected by research team members and entered into a Microsoft Excel spreadsheet, including (1) attending the initial assessment session (yes or no), and (2) attending the midintervention booster session (yes or no).

### Statistical Analysis

All statistical analyses were performed in the SPSS (version 28.0.1; IBM Corp). Given multiple statistical comparisons were conducted, a Bonferroni correction was applied to adjust for type I error inflation [55]. Missing data (% of missing items <0.001) were handled using single-value imputation. Sociodemographic and baseline clinical characteristics and user engagement data for all participants are reported using descriptive statistics. Absolute proportions are reported for categorical variables and means and SDs are reported for continuous variables. Chi-square tests were used to examine differences between adherers and nonadherers and fathers and mothers concerning categorical data. Effect sizes (Cohen w) were calculated to determine the magnitude of difference between the groups for categorical variables. Cohen w rule of thumb cutoffs was adopted: 0.1-0.3, indicating a small difference; 0.3-0.5, a moderate difference; and >0.5, a large difference [56]. Independent-sample *t* tests were used to examine differences regarding continuous variables.

### Ethical Considerations

This study was approved by the Regional Ethical Review Board in Uppsala, Sweden (Dnr: 2017/527), with an amendment obtained from the Swedish Ethical Review Authority (2019-03083). The research was conducted following the Helsinki Declaration and relevant Swedish regulations for human subject research. Parents interested in participating provided informed consent via the Portal. Data collected for the study were pseudonymized to ensure participant confidentiality. Participants were not compensated for their participation in the study. Participation was voluntary, and participants could withdraw at any time.

## Results

### Intervention Flow

**Figure 2:**
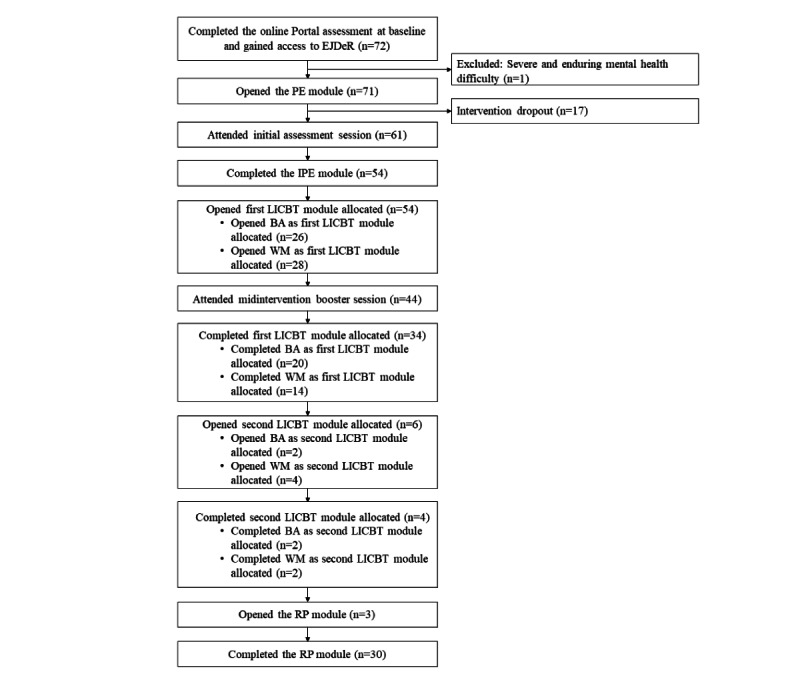
Flow of participants through the EJDeR intervention. BA: behavioral activation; EJDeR: intErnetbaserad sJälvhjälp för förälDrar till barn som avslutat en behandling mot canceR; IPE: introduction and psychoeducation; LICBT: low-intensity cognitive behavioral therapy; PE: psychoeducation; RP: relapse prevention; WM: worry management. Two participants were provided access to the RP module without completing a LICBT module, due to e-therapist error.

The flow of participants through the intervention is illustrated in Figure 2. In total, 77% (20/26) of participants starting with BA as the first LICBT module allocated completed the module versus 50% (14/28) starting with WM.

### User Engagement With the EJDeR Intervention

Participants spent an average of 205.36 (SD 154.64) minutes working with EJDeR, logging in an average of 19.99 (SD 14.95) times over 76.31 (SD 31.49) days. Participants opened a mean number of 2.3 (SD 0.92) modules and completed an average of 1.65 (SD 1.31) modules (Table 1).

**Table 1:** User engagement with the EJDeR intervention (N=71).

User engagement (log-data on the Portal)	Mean (SD)	Range
Minutes working with EJDeR^a^	205.36 (154.64)	8.59-611.74
Number of log-ins	19.99 (14.95)	1-72
Number of days using EJDeR	76.31 (31.49)	0-126
Number of written messages sent to e-therapists	8.52 (7.56)	0-33
Number of written messages sent to participants	28.79 (16.26)	0-74
Total number of modules opened	2.30 (0.92)	1-4
Total number of modules completed	1.65 (1.31)	0-4
Total homework exercises submitted	2.70 (2.78)	0-11

^a^EJDeR: intErnetbaserad sJälvhjälp för förälDrar till barn som avslutat en behandling mot cancer.

### Sociodemographic Between Intervention Adherers and Nonadherers

There were no significant differences between intervention adherers and nonadherers by sociodemographic characteristics (Table 2).

**Table 2:** Sociodemographic characteristics for total sample (N=71) and by adherers (n=34) and nonadherers (n=37) to the minimum treatment dose for the EJDeR intervention^a^.

Sociodemographic characteristics			Total study sample (N=71)				Adherers (n=34)					Nonadherers (n=37)					*P* value (effect size)^b^
			n (%)^c^		Range		n (%)		Range		n (%)			Range			
Age (years), mean (SD)			42.73 (7.02)		26-62		41.71 (5.30)		33-59		43.68 (8.25)			26-62		.24 (*d*=0.28)	
**Sex**																.99 (w=0.12)	
	Female	46 (65)		—^d^		22 (65)		—		24 (65)			—				
	Male	25 (35)		—		12 (35)		—		13 (35)			—				
**Relationship status**																.41 (w=0.05)	
	Partner	60 (85)		—		30 (88)		—		30 (81)			—				
	Single	11 (15)		—		4 (12)		—		7 (19)			—				
**Highest level of education**																.74 (w=0.09)	
	Lower secondary	1 (1)		—		0 (0)		—		1 (3)			—				
	Upper secondary	13 (18)		—		5 (15)		—		8 (22)			—				
	Postsecondary nontertiary	3 (4)		—		1 (3)		—		2 (5)			—				
	Tertiary	52 (73)		—		27 (79)		—		25 (68)			—				
	PhD	2 (3)		—		1 (3)		—		1 (3)			—				
**Employment status**																.90 (w=0.11)	
	Employed	63 (89)		—		30 (88)		—		33 (89)			—				
	Unemployed	8 (11)		—		4 (12)		—		4 (11)			—				
Number of children, mean (SD)			2.28 (0.78)		1-5		2.12 (0.69)		1-5		2.43 (0.84)			1-4		.09 (*d*=0.40)	
**Housing situation**																.07 (w=0.01)	
	Rental	7 (10)		—		2 (6)		—		5 (14)			—				
	Apartment ownership	17 (24)		—		6 (18)		—		11 (30)			—				
	House ownership	44 (62)		—		26 (76)		—		18 (49)			—				
	Other	3 (4)		—		0 (0)		—		3 (8)			—				
**Region of birth**																.18 (w=0.02)	
	Nordic countries^e^	59 (83)		—		31 (91)		—		28 (76)			—				
	Asia	6 (9)		—		3 (9)		—		3 (8)			—				
	Europe (excluding Nordic countries)	5 (7)		—		0 (0)		—		5 (14)			—				
	Africa	1 (1)		—		0 (0)		—		1 (3)			—				
**Previous psychological treatment**																.57 (w=0.07)	
	Yes	38 (54)		—		17 (50)		—		21 (57)			—				
	No	33 (46)		—		17 (50)		—		16 (43)			—				
**Physical health problems**																.61(w=0.07)	
	Yes	23 (32)		—		10 (29)		—		13 (35)			—				
	No	48 (68)		—		24 (71)		—		24 (65)			—				
**Previous traumatic or difficult life event**																.67 (w=0.08)	
	Yes	57 (80)		—		28 (82)		—		29 (78)			—				
	No	14 (20)		—		6 (18)		—		8 (22)			—				

^a^Adherence to EJDeR was conceptualized as users engaging with the intervention in accordance with a priori–defined minimum treatment dose. Intervention adherers are therefore defined as those (1) attending the initial assessment session with an e-therapist, (2) completing the psychoeducation module and one low-intensity treatment module (ie, behavioral activation or worry management), and (3) attending the midintervention booster session with an e-therapist.

^b^A Bonferroni correction was applied based on 11 tests resulting in an α of .005.

^c^Percentages may not add to 100 due to rounding.

^d^Not applicable.

^e^Nordic countries represented in the study sample include Denmark, Finland, Norway, and Sweden.

### Depression and Anxiety Scores Between Intervention Adherers and Nonadherers at Baseline

Nonadherers who opened BA as the first LICBT module allocated had a significantly higher level of depression symptoms at baseline than adherers (*P*=.04); however, this finding was no longer significant after applying a Bonferroni correction (α=.008). There were no other significant differences between intervention adherers and nonadherers by depression and anxiety score at baseline (Table 3).

**Table 3:** Depression and anxiety scores at baseline for total sample (N=71) and by adherers (n=34) and nonadherers (n=37) to the minimum treatment dose for the EJDeR intervention^a^.

Depression and anxiety scores at baseline	Total study sample (N=71)		Adherers (n=34)			Nonadherers (n=37)			*P* value (effect size)^b^
	n	Mean (SD)	n	Mean (SD)	n		Mean (SD)		
PHQ-9^c^ for all participants	71	6.55 (5.05)	34	6.24 (4.65)	37		6.84 (5.43)	.62 (d=0.12)	
PHQ-9 for participants who opened BA^d^ as first LICBT^e^ module^f^	26	8.31 (6.03)	20	7.00 (5.49)	6		12.67 (6.15)	.04 (d=0.97)	
PHQ-9 for participants who opened WM^g^ as first LICBT module^f^	28	4.46 (3.01)	14	5.14 (2.96)	14		3.79 (3.02)	.24 (d=–0.45)	
GAD-7^h^ for all participants	71	5.92 (4.53)	34	6.06 (4.25)	37		5.78 (4.83)	.80 (d=–0.06)	
GAD-7 for participants who opened BA as first LICBT module^f^	26	6.85 (5.09)	20	5.90 (4.62)	6		10.0 (5.73)	.08 (d=0.79)	
GAD-7 for participants who opened WM as first LICBT module^f^	28	5.11 (3.75)	14	6.29 (3.81)	14		3.93 (3.41)	.10 (d=–0.65)	

^a^Adherence to EJDeR was conceptualized as users engaging with the intervention in accordance with a priori–defined minimum treatment dose. Intervention adherers are therefore defined as those (1) attending the initial assessment session with an e-therapist, (2) completing the psychoeducation module and 1 low-intensity treatment module (ie, behavioral activation or worry management), and (3) attending the midintervention booster session with an e-therapist.

^b^A Bonferroni correction was applied based on 6 tests resulting in an α of .008.

^c^PHQ-9: 9-item Patient Health Questionnaire.

^d^BA: behavioral activation.

^e^LICBT: low-intensity cognitive behavioral therapy.

^f^Total study sample based on those gaining access to a low-intensity cognitive behavioral therapy module.

^g^WM: worry management.

^h^GAD-7: 7-item Generalized Anxiety Disorder scale.

### User Engagement Between Intervention Adherers and Nonadherers

There was a significant difference between adherers and nonadherers by first LICBT module allocated (*P*=.04); however, this finding was no longer significant after applying a Bonferroni correction (α=.007). Of the 34 adherers who opened the first LICBT module allocated, 59% (n=20) opened BA as the first LICBT module allocated and 41% (n=14) opened WM. Of the 20 nonadherers who opened the first LICBT module allocated, 30% (6) opened BA as their first LICBT module allocated and 70% (14) opened WM.

The number of minutes working with EJDeR (*P*≤.001), number of log-ins (*P*≤.001), number of days using EJDeR (*P*≤.001), number of written messages sent to e-therapists (*P*≤.001), number of written messages sent to participants (*P*≤.001), and total number of homework exercises submitted (*P*≤.001) were significantly higher among intervention adherers compared with nonadherers. There were no significant differences between adherers and nonadherers related to the other user engagement variables (Table 4).

**Table 4:** User engagement with the EJDeR intervention by adherers (n=34) and non-adherers (n=37) to the minimum treatment dose for the EJDeR intervention^a^.

Measure of user engagement (log-data on the Portal)		Adherers (n=34)		Nonadherers (n=37)			*P* value (effect size)^b^
		Mean (SD)	Range	Mean (SD)	Range		
Minutes working with EJDeR^c^		325.82 (126.61)	142.41-611.74	94.67 (74.21)	8.59-282.95	<.001 (d=2.23)	
Number of log-ins		31.32 (12.64)	5-72	9.57 (7.42)	1-29	<.001 (d=2.10)	
Number of days using EJDeR		91.47 (13.76)	57-126	62.38 (36.58)	0-126	<.001 (d=1.05)	
Number of written messages sent to e-therapists		12.88 (7.64)	2-33	4.51 (4.79)	0-17	<.001 (d=1.31)	
Number of written messages sent to participants		40.21 (12.09)	24-74	18.30 (11.98)	0-47	<.001 (d=1.82)	
**First LICBT^d^** **module allocated opened**						.04 (w=0.01)	
	Opened BA^e^, n (%)	20^f^ (59)	—^g^	6^h^ (30)	—		
	Opened WM^i^, n (%)	14^f^ (41)	—	14^h^ (70)	—		
Total homework exercises submitted		4.79 (2.53)	0-11	0.78 (1.13)	0-4	<.001 (d=2.05)	

^a^Adherence to EJDeR was conceptualized as users engaging with the intervention in accordance with a priori defined minimum treatment dose. Not all user engagement variables listed in Table 1 are included in this analysis as some measures of user engagement, that is, number of modules opened, attended initial assessment session, attended mid-intervention booster session, inform the minimum treatment dose.

^b^A Bonferroni correction was applied based on 7 tests resulting in an α of .007.

^c^EJDeR: intErnetbaserad sJälvhjälp för förälDrar till barn som avslutat en behandling mot canceR.

^d^LICBT: low-intensity cognitive behavioral therapy.

^e^BA: behavioral activation.

^f^number of adherers who opened the first low-intensity cognitive behavioral therapy module allocated (n=34).

^g^Not applicable.

^h^number of nonadherers who opened the first low-intensity cognitive behavioral therapy module allocated (n=20).

^i^WM: worry management.

### User Engagement Between Fathers and Mothers

There were no significant differences between fathers and mothers related to user engagement variables (Table 5).

**Table 5:** User engagement with the EJDeR intervention^a^ by fathers (n=25) and mothers (n=46).

Measure of user engagement					Fathers					Mothers					*P* value (effect size)^b^
					Statistical value		Range		Statistical value			Range			
**Log-data on the Portal**															
	Minutes working with EJDeR^c^, mean (SD)			185.53 (135.71)		18.67-546.51		216.14 (164.44)			8.59-611.74		.43 (*d*=–0.20)		
	Number of log-ins, mean (SD)			18.08 (14.05)		1-56		21.02 (15.46)			1-72		.43 (*d*=–0.20)		
	Number of days using EJDeR, mean (SD)			73.04 (33.79)		0-126		78.09 (30.41)			12-126		.52 (*d*=–0.16)		
	Number of written messages sent to e-therapists, mean (SD)			6.72 (5.93)		0-22		9.50 (8.20)			0-33		.14 (*d*=–0.39)		
	Number of written messages sent to participants, mean (SD)			26.52 (15.82)		0-66		30.02 (16.53)			7-74		.39 (*d*=–0.22)		
	Total number of modules opened, mean (SD)			2.28 (0.94)		1-4		2.30 (0.92)			1-4		.92 (*d*=–0.02)		
	Opened IPE^d^ module, n/N (%)			25/25 (100)		—^e^		46/46 (100)			—		—		
	**Completed IPE module, n/N (%)**												.57 (w=0.07)		
		Yes		19/25 (76)		—		32/46 (70)			—				
		No		6/25 (24)		—		14/46 (30)			—				
	**First LICBT^f^** **module allocated opened, n/N (%)**												.36 (w=0.04)		
		Opened BA^g^		8/20^h^ (40)		—		18/34^i^ (53)			—				
		Opened WM^j^		12/20^h^ (60)		—		16/34^i^ (47)			—				
	**Completed first LICBT module allocated**														
		**Completed BA, n/N (%)**											.39 (w=0.08)		
			Yes	7/8 (88)		—		13/18 (72)			—				
			No	1/8 (13)		—		5/18 (28)			—				
		**Completed WM, n/N (%)**											.45 (w=0.08)		
			Yes	5/12 (42)		—		9/16 (56)			—				
			No	7/12 (58)		—		7/16 (44)			—				
	**Opened RP^k^ module^l^** **, n/N (%)**												.03 (w=0.00)		
		Yes		8/20 (40)		—		24/34 (71)			—				
		No		12/20 (60)		—		10/34 (29)			—				
	**Completed RP module, n/N (%)**												.40 (w=0.07)		
		Yes		8/8 (100)		—		22/24 (92)			—				
		No		0/8 (0)		—		2/24 (8)			—				
	**Total homework exercises submitted, mean (SD)**			2.52 (2.52)		0-9		2.80 (2.94)			0-11		.68 (*d*=–0.10)		
**Data collected outside the Portal**															
	**Attended initial assessment session, n/N (%)**												.73 (w=0.09)		
		Yes		21/25 (84)		—		40/46 (87)			—				
		No		4/25 (16)		—		6/46 (13)			—				
	**Attended midintervention booster session, n/N (%)**												.80 (w=0.09)		
		Yes		16/25 (64)		—		28/46 (61)			—				
		No		9/25 (36)		—		18/46 (39)			—				

^a^Adherence to EJDeR was conceptualized as users engaging with the intervention in accordance with a priori–defined minimum treatment dose. Not all user engagement variables listed in Table 1 are included in this analysis as some measures of user engagement, that is, number of modules opened, attended initial assessment session, attended midintervention booster session, and inform the minimum treatment dose.

^b^A Bonferroni correction was applied based on 15 tests resulting in an α of .003.

^c^EJDeR: intErnetbaserad sJälvhjälp för förälDrar till barn som avslutat en behandling mot canceR.

^d^IPE: introduction and psychoeducation.

^e^Not applicable.

^f^LICBT: low-intensity cognitive behavioral therapy.

^g^BA: behavioral activation.

^h^Number fathers who opened the first low-intensity cognitive behavioral therapy module allocated (n=20).

^i^Number mothers who opened the first low-intensity cognitive behavioral therapy module allocated (n=34).

^j^WM: worry management.

^k^RP: relapse prevention.

^l^Two participants were provided access to the RP module without completing a low-intensity cognitive behavioral therapy module due to e-therapist error.

## Discussion

### Principal Findings

This study aimed to (1) describe user engagement with the EJDeR intervention, (2) examine whether sociodemographic and baseline clinical characteristics differed between adherers and nonadherers, (3) examine whether depression and anxiety scores differed between adherers and nonadherers at baseline, (4) examine whether user engagement differed between adherers and nonadherers, and (5) examine whether user engagement differed between fathers and mothers. Overall, 48% (34/71) of participants adhered to the minimum treatment dose. In total, 77% (20/26) of participants starting with BA as the first LICBT module allocated completed the module versus 50% (14/28) starting with WM. There were no significant differences between intervention adherers and nonadherers related to sociodemographic characteristics. Nonadherers who opened BA as the first LICBT module allocated had a significantly higher level of depression symptoms at baseline than adherers (*P*=.04); however, after applying a Bonferroni correction (α=.008), this finding was no longer significant. There were no other significant differences between intervention adherers and nonadherers by depression and anxiety score at baseline (Table 3). There was a significant difference between adherers and nonadherers by first LICBT module allocated (*P*=.04) however this finding was no longer significant after applying a Bonferroni correction (α=.007). Of the 20 nonadherers who opened the first LICBT module allocated, 30% (n=6) opened BA, and 70% (n=14) opened WM as their first LICBT module allocated. The number of log-ins, written messages sent to e-therapists, written messages sent to participants, and homework exercises submitted were significantly higher among intervention adherers than nonadherers. There were no significant differences between fathers and mothers related to user engagement variables.

### User Engagement and Adherence

Importantly, adherence differed by the first LICBT module allocated, with 30% (6/20) of nonadherers opening BA versus 70% (14/20) opening WM. One potential explanation for this finding is that BA interventions are simple, straightforward, and easy to comprehend [57,58] and, therefore, may be particularly well-suited to digital delivery. Conversely, the WM module included 2 treatment components [45,46] (problem-solving and worry time), which may have been perceived as more complex and challenging for participants. A recent study examining engagement with a mobile phone app to deliver WM found that 84% (803/956) of users did not achieve the minimum treatment dose [59]. In addition, the LICBT module BA is designed to overcome sources of negative reinforcement and increase engagement with pleasurable, routine, and necessary activities in a structured and graded way. BA may therefore result in more immediate and tangible rewards (eg, improved mood and increased engagement in valued activities) than WM. Indeed, BA is associated with sudden therapeutic gains [60], and a recent component network meta-analysis on iCBT for depression found stronger evidence for BA as an effective intervention component compared with other techniques, for example, cognitive restructuring and problem-solving [61].

However, differences in adherence by the first LICBT module allocated may also reflect the nonrandomized nature of module allocation. Module allocation was informed by the participant’s main presenting difficulties as assessed by the e-therapist, with a collaborative decision made with the participant to first start with BA for depression or WM for GAD. As such, the participant’s presenting difficulties, rather than module content alone, may have contributed to differences in adherence [62,63]. Overall, baseline clinical characteristics (depression and anxiety scores) did not differ between intervention adherers and nonadherers, similar to recent studies in other populations [64,65]. However, nonadherers who opened BA as the first LICBT module had a higher level of depression symptoms at baseline compared with adherers, although this finding was no longer significant after applying a Bonferroni correction. There were no other significant differences between intervention adherers and nonadherers by baseline clinical characteristics. The finding that nonadherers who opened BA as the first LICBT module had a higher level of depression symptoms at baseline is somewhat counterintuitive, given the effects of iCBT interventions are larger in those with moderate to severe depression [5]. However, other research has found higher levels of depressive symptoms at baseline to be associated with lower iCBT adherence [66,67] and higher rates of dropout from blended iCBT interventions [68]. Other studies have also found baseline anxiety symptoms to be an important predictor of adherence [69]. While this finding should be treated with caution, given small sample sizes and lack of power, symptoms at baseline may have influenced participants’ engagement with and adherence to specific modules, highlighting a potential need to consider baseline characteristics when allocating digital intervention components, for example, specific therapeutic techniques [63,70]. Future research with large sample sizes is warranted to explore the association between baseline clinical characteristics and intervention engagement and adherence.

Importantly, adherers had good levels of engagement with intervention components that were not related to the minimum treatment dose, for example, minutes working with the intervention, number of log-ins, number of written messages sent to e-therapists, number of written messages sent to participants, and the total number of homework exercises submitted. Examining user engagement variables not included in the minimum treatment dose by adherers and nonadherers helped provide a more nuanced understanding of how participants engaged (or not) with the intervention. Defining and measuring adherence to digital interventions in a way that accurately reflects user engagement is challenging, and there is a lack of universally accepted definitions of adherence [23]. Definitions typically vary depending on the intervention’s structure, ranging from module completion to log-in frequency to provide evidence of meaningful engagement with therapeutic components [71].

Indeed, defining user engagement and adherence in digital interventions is complex [72] and log-data tracking alone may not indicate a lack of intervention engagement, as participants may engage in intervention techniques offline, leading to higher levels of engagement than recorded by user action logging [64]. Furthermore, participants may have received an active treatment dose before reaching the a priori–determined minimum treatment dose, underscoring the complexities of conceptualizing adherence to digital interventions [18]. There have been calls to move beyond measuring user engagement using only behavioral tools (ie, system usage) and to measure emotional and cognitive engagement components of engagement [26]. For example, user engagement may include the extent of usage (eg, measured by user action logging) and subjective experience (eg, affect, attention, and interest) [20, 25]. In this study, we focused on measures of behavior within the Portal and did not measure subjective experience. Consequently, our findings may not fully capture the multifaceted nature of how participants may have engaged in the intervention and its associated factors. In our planned external pilot RCT, we will include additional measurements of user engagement beyond system usage data, such as validated self-report questionnaires [73,74] that measure affect, behavior, and cognition. However, there remains a need to develop ways of better defining, measuring, and differentiating between the different components of engagement in digital interventions [75].

The provision of support and guidance is another important factor potentially related to intervention adherence [28]. In this study, e-therapists sent an average of 40.21 (SD 12.09) written messages to adheres versus 18.30 (SD 11.98) to nonadherers. Personalized support and guidance are associated with improved retention [61] and effectiveness [5,6] in iCBT interventions. Our findings suggest additional consideration may be needed to train e-therapists to provide personalized support and motivate and encourage nonadherers to improve future intervention adherence. Future research could also look at providing an automated guidance function, for example, through a chatbot, to enhance adherence further [76].

No sociodemographic characteristics differed between intervention adherers and nonadherers. Furthermore, there were no significant differences between fathers and mothers regarding user engagement variables. The wider iCBT literature suggests mixed findings regarding the association between sociodemographic characteristics and adherence [77,78]. Some studies suggest that women and individuals with higher education levels are more likely to adhere to iCBT interventions compared with men and those with lower education levels [21]. However, conflicting results and lack of consistent associations limit the ability to conclude sociodemographic predictors of adherence [77].

### Implications

Findings build upon our previously published results from ENGAGE [31]. Analysis of usage data provides a more detailed and nuanced picture of how participants interacted with the intervention. This analysis also identifies key factors associated with engagement and adherence. One important finding is the difference in adherence based on the first LICBT module allocated, with 70% (14/20) of nonadherers who opened their first LICBT module opening WM versus 30% (6/20) of nonadherers opening BA. This suggests early exposure to potentially challenging and complex content may cause users to disengage. While developed using persuasive systems design elements [48,49], for example, tunneling, tailoring, rehearsal, and liking, future adaptations to the EJDeR intervention will need to focus on improving navigation and usability, especially for WM. Flexibility and ease of use of digital interventions for mental health have been identified as effective engagement strategies [75]. Engaging in user-centered design processes, that is, involving users in future system development, may represent a way to improve intervention engagement and adherence [59].

In addition, the WM module’s dual-component structure (ie, including 2 LICBT techniques—problem solving for practical worries and worry time for hypothetical worries) may have resulted in higher cognitive load, which could be a barrier to engagement and adherence. Participants first allocated to the WM module may benefit from additional support and guidance from an e-therapist. For example, e-therapists could be trained to provide more personalized support and feedback [59], which may help minimize challenges and help users understand how to engage with each module effectively [68]. Using visual aids, videos, and immediate feedback can also make content more accessible and motivating, improving adherence and overall user experience [75,76]. Furthermore, acknowledging that module length and complexity can impact adherence, and intervention modifications could also include efforts to streamline module design to reduce cognitive load without compromising therapeutic content. Although parents and e-therapists came to a collaborative decision to allocate the LICBT module to first work with, further intervention modifications could include providing more module-specific information, for example, a module preview, which may also facilitate starting with a module that meets their needs and preferences [79].

Finally, issues with engagement and adherence can arise due to digital interventions adopting a “one-size-fits-all” approach. Instead, interventions require careful tailoring to meet participants’ specific needs and context [80]. This is especially important when considering users of digital health interventions engage with these interventions in their own environment, whereby they may be experiencing multiple other demands [81]. iCBT interventions include user demands, such as time to engage in intervention (eg, reading the content, completing homework exercises, and attending support sessions). Lack of time is a common barrier to accessing psychological support reported by parents [82] and for parents of children treated for cancer, particularly [83]. Time was also the most frequently reported burden limiting participation in a group-based CBT videoconferencing program to support parents of children treated for cancer [84]. Parents may also experience reduced opportunities for privacy, further interfering with their ability to engage with the intervention. Other research exploring engagement with digital interventions found users with no children report higher intervention usage [85]. Other factors, including socioeconomic status, may influence how participants engage with interventions [14]. Furthermore, individual contextual factors such as digital literacy and access to technology can influence user engagement [86-88]. Despite the EJDeR intervention being developed alongside parent research partners [38], additional intervention tailoring may be required to further adapt the intervention to the specific needs and context of the population.

### Strengths and Limitations

Given that this study is a secondary analysis of data from a single-arm feasibility trial, the sample size is small, and analyses may be underpowered to detect statistically significant differences. Due to the small sample size, we could not conduct a regression or correlation analysis. Multiple statistical comparisons were conducted, increasing the risk of Type I error. However, this risk was minimized by applying a Bonferroni correction to reduce the likelihood of spurious results [55]. Most participants were female, had an education level higher than upper secondary school, and were born in a Nordic country. This may limit the generalizability of findings, especially considering Sweden is a diverse and multicultural society, with 21% (2,170,627/10,551,700) of the population being foreign-born [89], and there is a need to develop culturally responsive digital interventions to enhance acceptability and engagement in ethnic minority populations [90]. The research team defined intervention adherence a priori following a minimum treatment dose. This definition fails to consider that participants may have conducted intervention activities outside the Portal, that is, offline [59]. Some participants may have achieved their personal intervention goals early and stopped engaging with the intervention, and yet were classified as intervention nonadherers [12].

We also adopted an exploratory approach that aimed to examine a broad range of variables potentially associated with user engagement and adherence. We recognize that the exploratory analysis of log-data has significant limitations, for example, lack of transparency, difficulties with replication, and the risk of “data dredging” [29]. While we set specific research aims to inform our approach, we did not use a framework to underpin our analysis. Adopting the framework for Analyzing and Measuring Usage and Engagement Data (AMUsED) [29] to define meaningful usage variables and generate research questions related to factors such as the interventions’ design and theoretical underpinning would have strengthened our approach. Recognizing this limitation, we aim to adopt the AMUsED framework to inform usage analysis in our external pilot RCT to prepare for the design and conduct of a future superior RCT. Our exploratory approach also risked circular analysis, for example, examining user engagement variables associated with adherence may be considered circular given our definition of adherence is partially defined by intervention usage. However, as adherence (ie, the minimum treatment dose) was defined as attending sessions with the e-therapist and module completion, this does tell us much about how adherers versus nonadherers engaged with the Platform beyond these 2 metrics. For example, user engagement variables associated with frequency of use (eg, length of time working with EJDeR and number of log-ins) or engagement in intervention content such as homework exercises (eg, number of written homework exercises submitted) provided more nuanced information concerning how adherers versus nonadherers interacted with the intervention beyond the minimum treatment dose.

As a feasibility study, ENGAGE was not designed to examine the effectiveness of the EJDeR intervention, and we did not examine the association between intervention usage and clinical outcomes. This association will be examined in the planned future superiority RCT, should progression to a superiority RCT be warranted after conducting the external pilot RCT. The association between intervention usage and clinical outcomes will be used to further inform our definition of the minimum treatment dose by helping us to understand what optimal level of intervention usage can affect clinical outcomes [28].

Despite these limitations, our study has some important strengths. We examined the relationship between engagement and adherence, considering the therapeutic content of modules (eg, BA or WM) [59]. In addition, we used objective data (eg, log-data tracking on the Portal and data collected outside the Portal by members of the research team) to examine engagement, overcoming biases associated with self-reported usage data [91].

### Conclusions

Our findings provide important information concerning modifications to the EJDeR intervention to enhance user engagement and adherence, which may be related to intervention efficacy. Findings may also have broader applicability to improving user engagement in other iCBT interventions. Our findings suggest simple and straightforward LICBT techniques, such as BA, are well-suited to digital delivery. However, more complex LICBT techniques, such as WM, may need modification to improve ease of use and thus increase user engagement and adherence. Participants using WM or other more complex therapeutic techniques and those with a higher level of depression symptoms at baseline may need additional support from an e-therapist. Finally, engaging in user-centered design processes (ie, involving users in future system development [92]) may represent a way to improve intervention engagement and adherence [59] by improving navigation as well as further tailoring the intervention to the needs and context of parents of children treated for cancer.

## Abbreviations

AMUsED: Analyzing and Measuring Usage and Engagement Data
BA: behavioral activation
CBT: cognitive behavioral therapy
CONSORT: Consolidated Standards of Reporting Trials
EJDeR: internetbaserad självhjälp för föräldrar till barn som avslutat en behandling mot cancer or internet-based self-help for parents of children who have completed cancer treatment
GAD: generalized anxiety disorder
GAD-7: 7-item Generalized Anxiety Disorder scale
iCBT: internet-administered cognitive behavioral therapy
IPE: introduction and psychoeducation
LICBT: low-intensity cognitive behavioral therapy
PHQ-9: 9-item Patient Health Questionnaire
RCT: randomized controlled trial
RP: relapse prevention
U-CARE: Uppsala University Psychosocial Care Programme
WM: worry management

## Data Availability

The data that support the findings of this study are available from the corresponding author (LvE) upon reasonable request.
